# Histopathological analysis potential for unveiling hormone signaling in endocrine-related tumors

**DOI:** 10.1530/EO-24-0033

**Published:** 2024-08-29

**Authors:** Yasuhiro Miki, Erina Iwabuchi, Chihiro Inoue, Yuto Yamazaki, Takashi Suzuki

**Affiliations:** 1 Department of Anatomic Pathology, Tohoku University Graduate School of Medicine, Sendai, Japan; 2 Department of Pathology and Histotechnology, Tohoku University Graduate School of Medicine, Sendai, Japan; 3 Department of Pathology, Tohoku University Hospital, Sendai, Japan

**Keywords:** immunohistochemistry, *in situ* proximity ligation assay, southwestern histochemistry, steroid hormone

## Abstract

Elucidating the mechanisms of action of steroid hormones will contribute to the development of therapeutic strategies for hormone-dependent tumors. Recent advances in genetic engineering have revealed the complex and diverse mechanisms of steroid hormone signaling; however, these techniques are limited to *in vitro* or animal experiments. It is believed that verifying hormone signals elucidated using human pathological tissue specimens will directly aid in treatment and diagnosis. However, pathological tissue specimens are generally formalin-fixed paraffin-embedded (FFPE), and protein/gene analyses of FFPE tissues are limited. Protein detection using immunohistochemistry with specific antibodies in FFPE tissues is a classical technique essential for diagnosis and treatment decisions in various types of cancer. In steroid hormone signaling, the expression and localization of receptors, hormone-related enzymes, and proteins encoded by response genes can be clarified using immunohistochemistry. Although protein-protein interactions such as receptor dimers and DNA-binding proteins are mainly detected *in vitro*, they can be examined in FFPE tissues using *in situ* proximity ligation assays and southwestern histochemistry, respectively. Using these detection methods, including immunohistochemistry, it is possible to analyze each hormone signaling pathway in hormone-related tumors histopathologically. Although FFPE tissues still suffer from gene and protein denaturation, their advantages include the ability to retrospectively study target factors/signals and obtain spatial information through microscopy. This review describes a visualization method for elucidating steroid hormone signaling in hormone-dependent tumors using FFPE tissues.

## Introduction

Estrogen receptor (ER)-positive breast cancers grow when estrogen binds to ER in breast cancer cells. Therefore, ER-positive breast cancer is considered a typical hormone-related cancer. Endocrine therapies related to anti-estrogen signals for ER-positive breast cancer can be classified into those that target estrogen receptors and those that target estrogen synthesis based on their different mechanisms of action (Burstein2019, Rej *et al.* 2024). To determine whether breast cancer is estrogen dependent, ER immunohistochemistry is performed on pathological specimens. The results of this ER immunohistochemical analysis are considered when determining the indications for the endocrine therapies described above. As a type of anti-estrogen therapy, aromatase inhibitors are prescribed as first-line drugs in patients with ER-positive breast cancer. To understand the local synthesis of estrogen in breast cancer, measuring its concentration is necessary; however, this method is not easy, such as securing fresh frozen samples. Therefore, immunohistochemical detection of enzymes involved in estrogen synthesis is an alternative method. Although aromatase immunohistochemistry is not used as an indicator of aromatase inhibition in patients with ER-positive breast cancer, it can be used to determine the local synthesis of estrogen in several cancer types (Miki *et al.* 2007, Miki *et al.* 2010, Grindstad *et al.* 2016).

**Figure 1:**
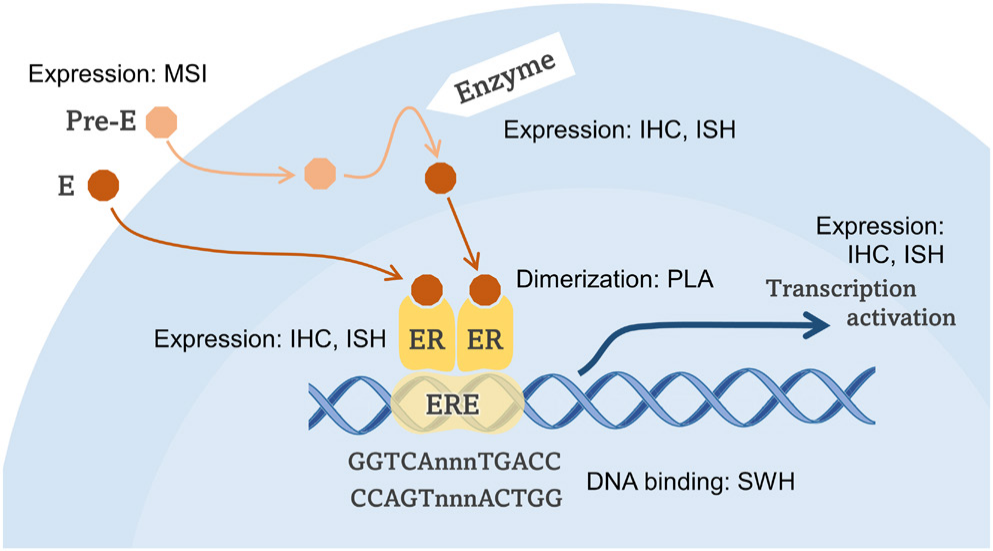
Estrogen receptor signals and corresponding histopathological analysis. Estrogen (E) binds to the estrogen receptors (ER) in the nucleus. Estrogen precursor hormones (pre-Es) are converted to estrogen by related enzymes (Enzyme) in the cytoplasm. Estrogen-bound receptors dimerize and bind to the estrogen receptor response element (ERE) upstream of the target gene to activate its transcription. IHC, immunohistochemistry; ISH, *in situ* hybridization; PLA, *in situ* proximity ligation assay; SWH, southwestern histochemistry; MSI, mass spectrometry imaging.

Many steroid hormones, including estrogen, induce specific effects by acting on their receptors in the cytoplasm or nucleus. For example, when estrogen enters a cell and binds to the ER in the nucleus, the receptor forms a dimer. Activated ER binds to DNA sequences called estrogen receptor response elements (EREs), which in turn induce or repress the transcription of target genes depending on other transcription factors and co-factors (Aranda & Pascual 2001). Figure 1 shows a typical estrogen-ER genomic signal. The following hormone signaling pathway process can be analyzed histologically: 1. expression of receptors; 2. detection of dimerized receptors; 3. receptor binding to DNA response elements; 4. expression of response genes; 5. localization of hormones. For gene transcriptional regulation by ligand-nuclear receptors, interacting with transcription factors that gather in the transcriptional activation domains (AF-1 and AF-2) of the receptors is important.

Cancer tissues are composed of not only cancer cells, but also various types of stroma, such as inflammatory cells, fibroblasts, and capillaries. Fibroblasts specific to cancer tissues are known as cancer-associated fibroblasts and are involved in regulating hormone signaling not only in typical hormone-dependent tumors, such as breast cancer and prostate cancer, but also in lung cancer (Inoue *et al.* 2023). Analyses that involve homogenizing whole tissues do not allow us to know from which cell the desired hormone signals originate; however, this becomes possible using pathological tissue specimens. Furthermore, retrospective cohort studies using formalin-fixed paraffin-embedded (FFPE) tissues may reveal the clinicopathological significance, including prognosis, of the hormonal signals of interest (Gaffney *et al.* 2018). However, dynamic information cannot be obtained from FFPE tissues, thus limiting the molecular biological elucidation of hormone action mechanisms. Although molecular biological analysis using FFPE tissue has been developed in recent years, the microscopic observation level for identifying cells has not yet been reached. Hormone receptor detection using immunohistochemistry is a typical hormone signal detection technique that uses pathological tissue specimens. Furthermore, by examining the expression of hormone-related enzymes and hormone response proteins, predicting hormone signaling in tumor tissues is possible. In this review, we describe techniques, such as immunohistochemistry, that can detect the hormone signals shown in Fig. 1 using pathological FFPE tissues.

## Detection of hormone receptors

Non-radioisotopic methods, such as enzyme immunoassay (EIA) and enzyme-linked immunosorbent assay (ELISA), have enabled the quantitative detection of steroid hormones and their receptors (Pousette *et al.* 1986). Fresh tissue or fresh frozen tissue homogenates are used for measurements using tissue-based EIA or ELISA, which means that these methods cannot reveal the localization of the receptor to be evaluated within the tissue. Moreover, ER scores assessed by immunohistochemistry were significantly correlated with ER measurements by EIA (Horiguchi *et al.* 2003). The immunohistochemical detection of cancer biomarkers that guide treatment has been well validated, and some of them, such as ER and progesterone receptor (PgR) in breast cancer, have been approved by the Food and Drug Administration (FDA) (O’Hurley *et al.* 2014). In both clinical and basic research, immunohistochemistry of hormone receptors using pathological specimens is considered an essential technique for understanding their localization, expression, and significance.

**Figure 2:**
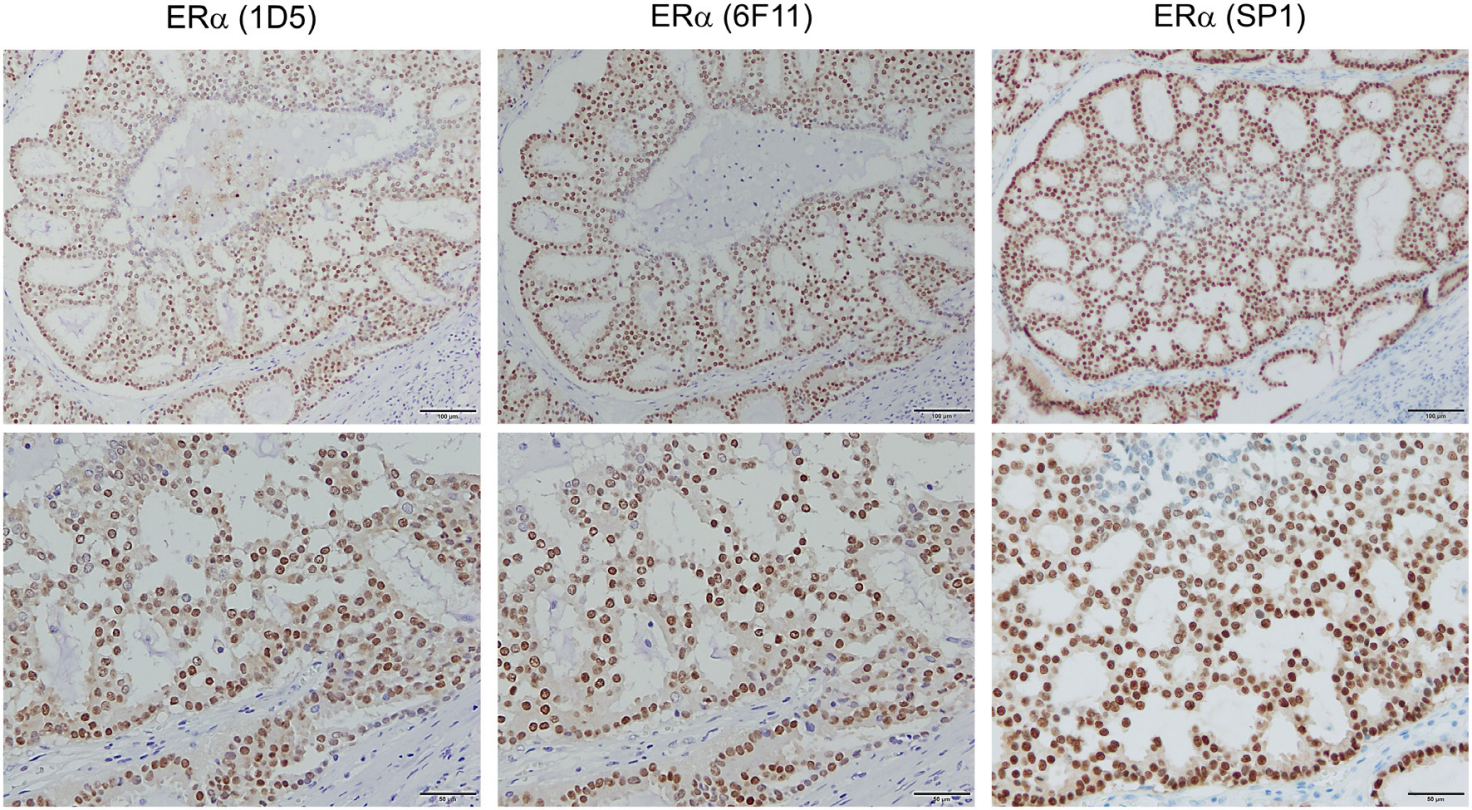
Immunohistochemistry of estrogen receptor in the breast cancer tissue. Immunohistochemistry of three types of anti-human estrogen receptor ⍺ (ER⍺) antibodies in invasive ductal carcinoma of the same case is shown. For immunostaining with SP1, an autostainer (VENTANA BenchMark ULTRA, Roche Diagnostics, Rotkreuz, Switzerland) and its kit (Ventana UltraView confirm ER (SP1), Roche Diagnostics) were used. 1D5, mouse monoclonal antibody; 6F11, mouse monoclonal antibody; SP1, rabbit monoclonal antibody. The upper panel shows low magnification (scale bar, 100 μm); the lower panel shows high magnification (scale bar, 50 μm).

The most important factor in antigen detection by immunohistochemistry is the primary antibody clone used. Bogina *et al.* (2012) compared the staining properties of pathological breast cancer specimens by immunohistochemistry using three types of monoclonal antibodies against ER. The results showed that the sensitivity of 1D5 was lower than that of SP1 and 6F11. Furthermore, the percentage of immunoreactive-positive cells was significantly higher in SP1 than in the other two, and in 6F11 than in 1D5. SP1 is an antibody derived from immunized rabbits, whereas 6F11 and 1D5 were obtained from immunized mice. Rabbit-derived monoclonal antibodies are considered to have a higher affinity for antigens than mouse-derived monoclonal antibodies (Vilches-Moure & Ramos-Vara 2005). SP1 recognizes the N-terminal aa 578–595, whereas 6F11 and 1D5 recognize the C-terminal aa 15–23 and aa 127–130, respectively (Kornaga *et al.* 2016). Furthermore, the characteristics of the antibody, i.e., the type of animal immunized and the epitope recognized, are potential factors affecting the immunohistochemistry results. Immunohistochemistry for ER in breast cancer using the three types of antibodies described above is shown in Fig. 2.

Androgen receptor (AR) is complicated, with a variety of genomic and protein variants. AR lacking the N-terminal domain, which has a molecular mass of 45 kDa (AR45), exerts an inhibitory response against androgens (Ware *et al.* 2014). Furthermore, variants with shortened C-termini, such as ARV567ES and ARV7, have been reported to be involved in castration resistance in prostate cancers (Ware *et al.* 2014). The immunogen of AR441, a mouse monoclonal AR antibody, is the N-terminal aa 299–315, so it cannot distinguish between wild-type AR, ARV567ES, and ARV7 and cannot even recognize AR45. Immunohistochemistry using rabbit and mouse monoclonal antibodies that recognize the AR N-terminus in TNBC showed that rabbit monoclonal antibody SP107 had a higher immunoreactivity positivity rate than mouse AR441 (Kumar *et al.* 2017). Recently, immunohistochemistry of the AR N-terminal (AR441) and C-terminal (SP107) in human heart tissue has been investigated (Eildermann *et al.* 2023); however, immunohistochemistry remains unclear in hormone-dependent cancers. Antibodies against AR-V7 are commercially available and have been used for *in vitro* analyses (Liu *et al.* 2014, Khatiwada *et al.* 2024), and immunohistochemistry using this antibody has also been reported in prostate and breast cancers (Qu *et al.* 2015, Hickey *et al.* 2015). The investigation of splicing variants in tissues using immunohistochemistry requires the use of carefully validated antibodies against the sequencing results.

Immunohistochemistry for nuclear antigens can be quantified by counting the number of positive nuclei, and statistical analyses can be performed. A labeling index or H-score is used for quantification (Crispino *et al.* 1989, Snead *et al.* 1993, Meyerholz & Beck 2018), and the counting location is an arbitrary field of the specimen or a hotspot where positive cells are clustered (Hida *et al.* 2015, Yamazaki *et al.* 2016). For the detection of proteins in tissues using immunohistochemistry, selecting a primary antibody based on its antigenic characteristics, which take into account the epitope, is important, as is the appropriate dilution concentration of the antibody. Further considerations for tissues include the tissue fixation method, fixation time, and antigen retrieval treatment, which greatly influence the results of immunohistochemistry (O’Hurley *et al.* 2014, Ivell *et al.* 2014).

## Detection of hormone-related enzymes

Hormone concentrations can be measured with high sensitivity using the insulin radioimmunoassay developed by Yalow & Berson (1959), which has been applied not only to insulin but also to peptide and steroid hormones. Subsequently, immunoradiometric assays were developed to measure trace amounts of hormones, and immunoassays using non-isotopic labels (chemiluminescence, fluorescence, and enzymes) were established. As the demand for measuring trace amounts of hormones, such as sex steroid hormones, to diagnose pubertal diseases in immature children has increased, hormone measurements using liquid chromatography-tandem mass spectrometry (LC-MS/MS) have become widely used (Moal *et al.* 2007, Rauh 2009). Even in the case of hormone-related cancers, trace hormone measurements provide useful information for both diagnosis and research, such as the pathophysiology of cancers and the effects of treatments (Koal *et al.* 2012, Choi 2021, Snaterse *et al.* 2023). To measure intratumoral/intratissular steroid hormone concentrations using the aforementioned methods, fresh frozen tissues are required. However, steroid hormones are lost in FFPE tissues owing to exposure to organic solvents during the preparation process; therefore, using them as samples for measurements is difficult.

**Figure 3:**
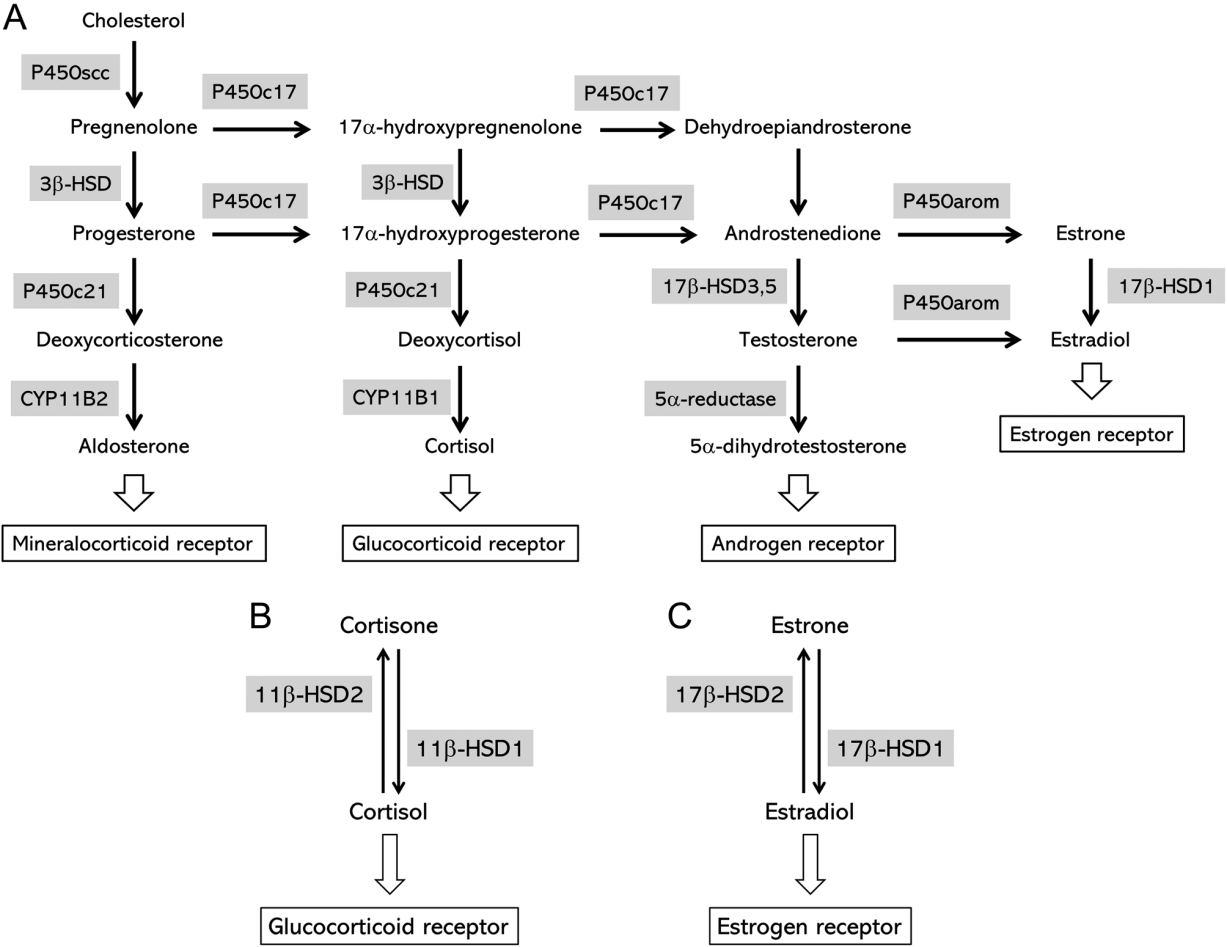
Steroid hormone biosynthesis pathway. A. The pathway by which various hormones are synthesized from cholesterol and the enzymes involved are shown. B. The conversion of cortisol/cortisone by 11β-HSDs is shown. C. The conversion of estradiol/estrone by 17β-HSDs is shown.

Steroid hormones are primarily produced from cholesterol in the blood through metabolism by several enzymes (Fig. 3A). Steroid hormones are produced in the adrenal cortex, gonads, and placenta, and their secretion is strictly and physiologically regulated by a feedback mechanism based on the hypothalamus-pituitary-steroid-producing gland axis. In the adrenal cortex, aldosterone is produced in the zona glomerulosa, and cortisol is produced in the zona fasciculata, with *CYP11B2* and *CYP11B1* being the enzymes involved in their final synthesis steps, respectively. The HISTALDO consensus recommends that the histopathological diagnosis of hyperaldosteronism should be based on morphology using hematoxylin and eosin staining of adrenal lesions and immunohistochemistry for *CYP11B2* to confirm the presence of autonomous aldosterone secretion in them (Williams *et al.* 2021). Therefore, intratumoral/intratissular steroid hormone concentrations can be indirectly predicted by detecting the expression of steroid hormone-related enzymes by immunohistochemistry using FFPE tissues.

We previously reported that intratumoral 5⍺-dihydrotestosterone (DHT) measured using LC-MS/MS was significantly associated with immunohistochemistry of 5⍺-reductase, the enzyme that converts testosterone into DHT, in breast and endometrial cancers (Suzuki *et al.* 2007, Tanaka *et al.* 2015). Cortisol, ultimately produced through *CYP11B1* catalysis, is inactivated into cortisone by 11β-hydroxysteroid dehydrogenase (HSD) 2 in the periphery. Cortisone is converted to its activated form, cortisol, by 11β-HSD1, and cortisol concentration in peripheral tissues is thought to be regulated by an enzymatic activity between 11β-HSD1 and 11β-HSD2 (Fig. 3B). Quantitative intratumoral cortisol using LC-MS/MS in endometrial cancer revealed that the hormone concentrations were higher in the 11β-HSD1-positive group than in the negative group, and in the 11β-HSD2-negative group compared with the positive group (Miki* et al.* 2023*a*,*b*). The relationship between intratumoral estradiol and DHT concentrations and the immunohistochemistry of their related enzymes were evaluated in nine breast cancer tissue samples (Takagi *et al.* 2010). In this report, estradiol concentrations were higher in the 5α-reductase negative group and the 17β-HSD2 negative group, which converts estradiol to estrone (Fig. 3C). The absence of 5α-reductase, which catalyzes the conversion of testosterone to 5α-DHT, resulted in the dominance of testosterone conversion to estradiol. Similarly, the absence of 17β-HSD2, which converts estradiol to estrone, led to increased estradiol levels. However, the immunoreactivities of several related enzymes, including aromatase, which significantly contributes to intratumoral estrogen concentrations, were not associated with estradiol concentrations in nine breast cancers (Takagi *et al.* 2010). As shown in Fig. 3, several enzymes are involved in steroid hormone biosynthesis in a cascading manner, suggesting that the expression of enzyme groups located in the middle of the cascade is unlikely to reflect the actual hormone concentration.

Immunohistochemistry for cytoplasmic antigens was evaluated based on the degree of staining intensity (e.g., negative, weakly positive, and strongly positive) and grouped based on the percentage of staining in the entire specimen (Suzuki *et al.* 2007, Tanaka *et al.* 2015, Meyerholz & Beck 2018). Population studies on clinicopathological significance, including the prognosis of hormone-related enzymes, can be performed retrospectively using FFPE tissues (Henry *et al.* 1991, Suzuki *et al.* 2007, Tanaka *et al.* 2015). Immunohistochemistry can also reveal the localization of expression, indicating which cell components (i.e., parenchymal and stromal cells) in cancer tissues express the enzyme (Miki *et al.* 2007, Miki *et al.* 2010). However, in this case, antibodies that have been well verified through pilot experiments, such as their relationship with measurements of intratumoral hormone concentrations, should be used.

## Detection of hormone-induced proteins

Steroid hormone receptors are ligand-dependent transcription factors that exert a variety of functions by regulating the expression of various downstream response genes located near their response elements. In some cases, the protein initially produced in the primary reaction exerts a direct biological effect. In others, the protein itself becomes a transcriptional regulatory protein and secondarily promotes protein transcription and translation. In the histological examination of receptors and proteins encoded by these response genes, immunohistochemistry has revealed relationships between ER and pS2/pNR-2/TFF1 in breast cancer (Henry *et al.* 1991, Koerner *et al.* 1992, Suzuki *et al.* 2004) and between AR and prostate-specific antigen in prostate cancer (Zhang *et al.* 1998, Ogreid *et al.* 1999). Histological analysis cannot be used to assess whether the detected protein is produced primarily or secondarily during a hormonal response. However, immunohistochemistry is useful for understanding the relationship and colocalization of proteins with receptors or hormone-related enzymes.

Immunohistochemistry has revealed a positive relationship between the ER and proteins that have ERE in their encoded gene promoters, such as *EBAG9*, *cyclin D1*, and *PgR* in breast cancer (Suzuki *et al.* 2004). Furthermore, estrogen-responsive finger protein (*efp*), a typical ER primary response gene, had a positive relationship with ERα in breast cancer (Suzuki *et al.* 2005, Ko *et al.,* 2014) and with both ERα and ERβ in ovarian cancer (Sakuma *et al.* 2005). However, cases of unrelated ER and *efp* immunoreactivity in breast cancer have been reported (Thomson *et al.* 2001). While it is necessary to check the clinical pathological background of the subjects in these reports, differences in immunohistochemical evaluation and methods are suggested to be the cause of the discrepancy in results.

**Figure 4:**
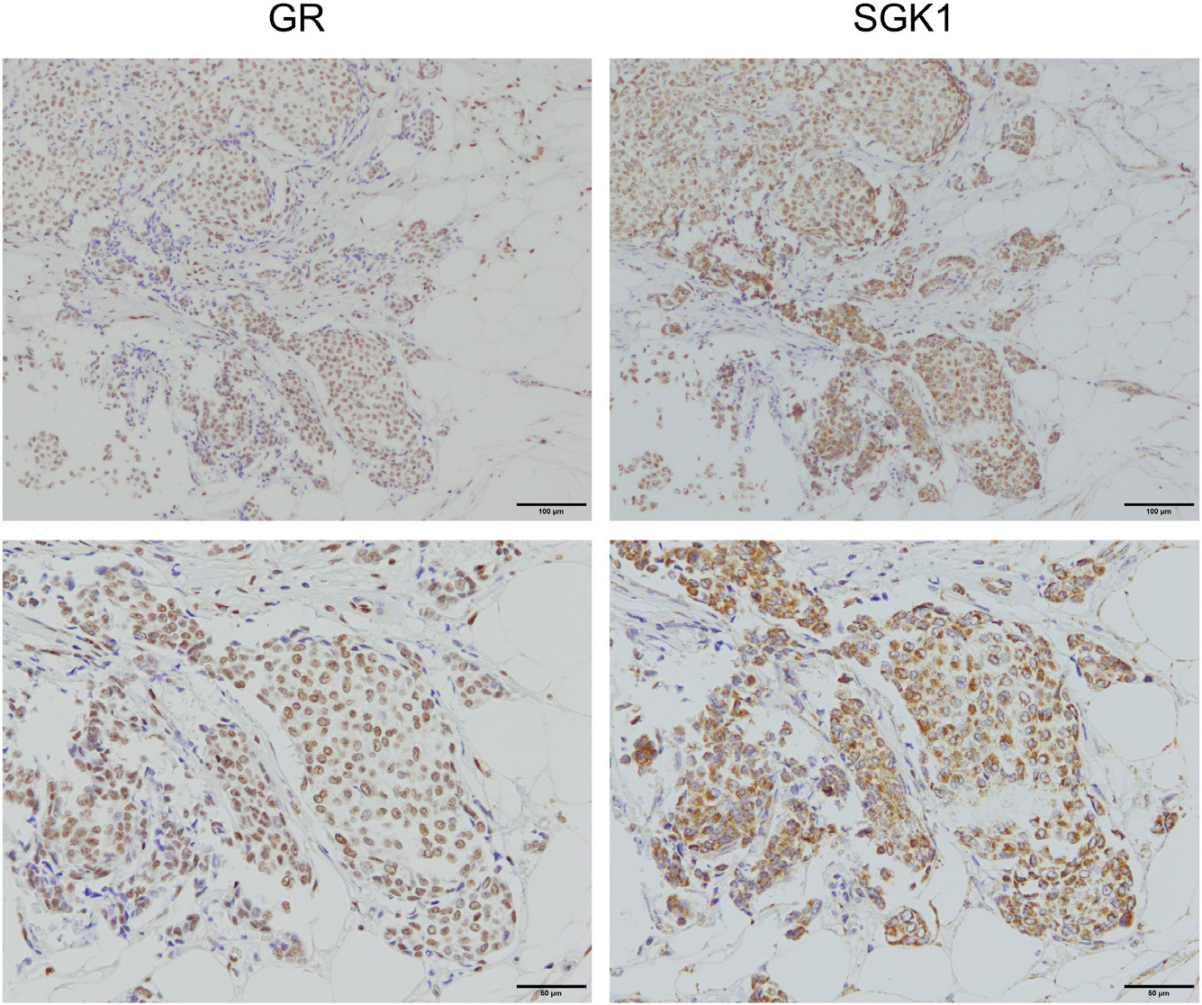
Immunohistochemistry of glucocorticoid receptor and serum/glucocorticoid-regulated kinase 1 in breast cancer. In invasive ductal carcinoma, immunoreactivity was observed for the glucocorticoid receptor (GR) in the nucleus of cancer cells and for serum/glucocorticoid-regulated kinase 1 (*SGK1*) in the cytoplasm of the same region.

**Figure 5:**
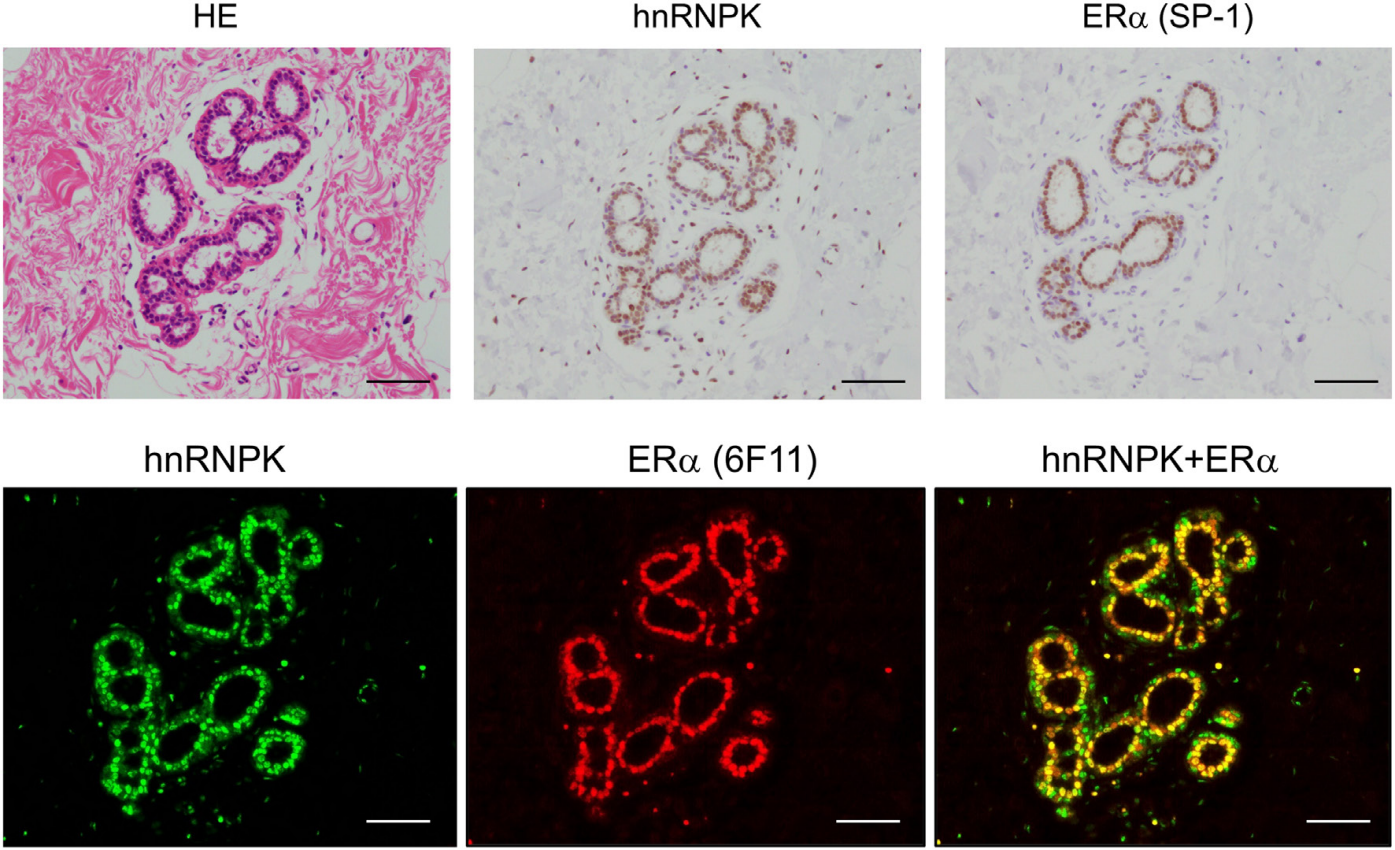
Immunohistochemistry of estrogen receptor α and HNRNPK in normal mammary epithelial cells. Immunohistochemistry of estrogen receptor α (ERα) and HNRNPK in normal breast ductal epithelial cells associated with invasive ductal carcinoma is shown. The upper panels show hematoxylin-eosin staining (HE) and immunohistochemistry (bright field) of ERα (rabbit monoclonal SP1) and HNRNPK (rabbit monoclonal GTX61456). The lower panels show ERα (mouse monoclonal 6F11) and HNRNPK (GTX61456) immunofluorescence histochemistry and their merged images (HNRNPK+ERα).

Figure 4 shows the immunolocalization of the glucocorticoid receptor (GR) and serum/glucocorticoid-regulated kinase-1 (*SGK1*) in breast cancer cells. *SGK1* has been identified as a response gene induced by serum and glucocorticoids in rat mammary tumor cells and is a typical GR response gene (Lang *et al.* 2006). Both GR and *SGK1* were expressed in the same breast cancer lesions (Fig. 4). Furthermore, HNRNPK has an ERE in its promoter region (Nagai *et al.* 2004) and is induced by estrogen in ER-positive breast cancer cell lines (Iwabuchi *et al.* 2021). The HNRNPK index in endometrial cancer tissues was significantly positively correlated with intratumoral estrogens (estrone and estradiol) measured by LC-MS/MS but was unrelated to androgens (testosterone and DHT) (Miki *et al.* 2022). In addition, public database analysis revealed that HNRNPK gene expression was positively correlated with ERα gene expression but not ERβ, and the HNRNPK gene high expression group had a significantly better prognosis in endometrial cancer patients. Figure 5 shows the immunofluorescence histochemistry of HNRNPK in normal mammary epithelial cells. Moreover, HNRNPK was expressed in the same cells as ERα-positive cells. Expression analysis of HNRNPK in breast cancer FFPE tissue revealed that HNRNPK immunoreactivity was significantly higher in the ERα immunohistochemical index and significantly lower in the cancer proliferation marker Ki-67 index (Iwabuchi *et al.* 2021). In the breast cancer cell line MCF-7, *in situ* proximity ligation assays (PLA) (see next section) showed that the HNRNPK protein induced by the ER pathway interacts with the ERα protein, suggesting that HNRNPK modulates ER function through protein-protein interactions (Iwabuchi *et al.* 2021).

## Visualization of hormone receptor signals: detection of protein-protein interaction

**Figure 6:**
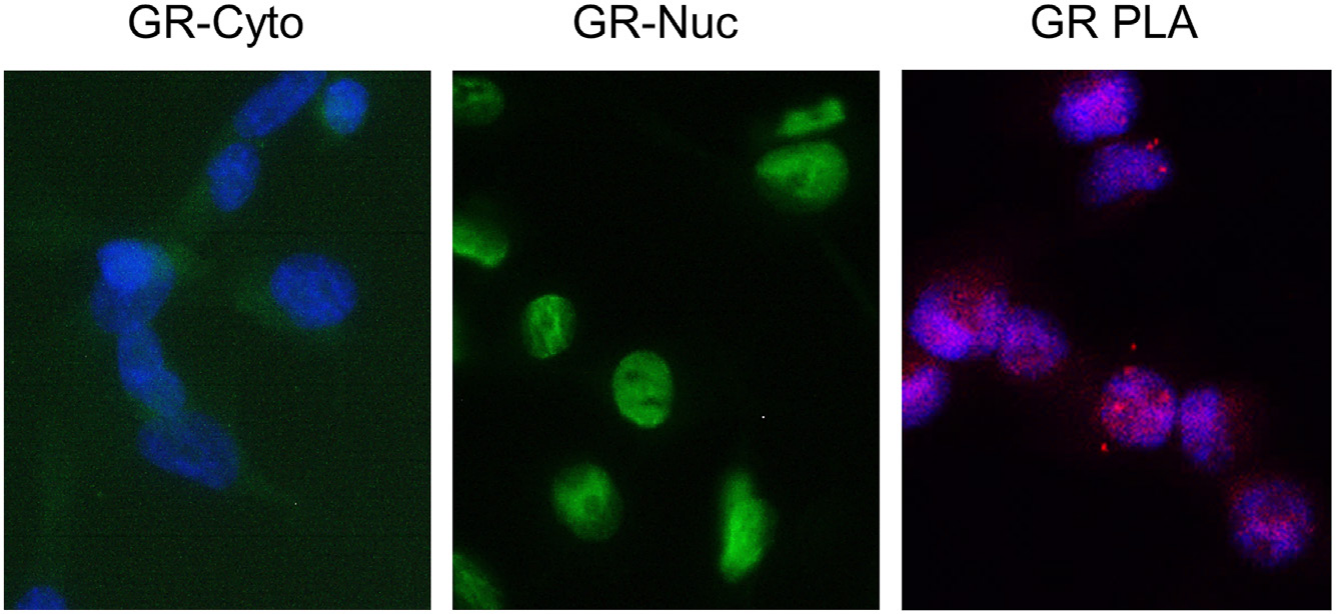
Subcellular localization of the glucocorticoid receptor in the breast cancer cell line. Immunofluorescence cytochemistry and proximity ligation assay (PLA) of glucocorticoid receptor (GR) in MDA-MB-231 cells are shown. Under hormone-depleted conditions, GR localizes to the cytoplasm (GR-Cyto), and dexamethasone addition localizes it to the nucleus (GR-Nuc). PLA of GR (GR-PLA) in dexamethasone-treated MDA-MB-231 cells is shown. Rabbit monoclonal D6H2L antibody was used for immunofluorescence histocytochemistry, and D6H2L and mouse monoclonal antibody (NCL-GCR) were used for PLA.

Protein-protein interactions (PPI) are ubiquitous in biological signaling systems and are a fundamental reaction in the activation of hormone signals. The hormone receptor, which binds to the ligand and then to DNA, acts as a large complex that binds to transcriptional co-factors and modulates transcription by altering the structure of chromatin. Some hormone receptors wait for ligands to enter the cytoplasm. The GR binds to proteins called chaperones, such as HSP90 and p23, in the cytoplasm, and when a ligand binds to GR, the GR separates from the chaperone and translocates into the nucleus (Echeverría *et al.* 2009). The ligand-GR binds to the glucocorticoid response element of the target gene. Figure 6 shows the nuclear translocation of GR in the breast cancer cell line MDA-MB-231. GR was weakly expressed in the cytoplasm under hormone deprivation (72 h) but was clearly detected in the nucleus when dexamethasone (10 nM, 30 min) was added. Immunohistochemistry allows the expression of receptors and transcription factors to be visualized in pathological specimens at the protein level. However, it is not possible to determine their PPIs. Visualizing and evaluating PPI in pathological tissues will help clarify the significance of more activated hormone signals.

PLA is a tool that combines antibodies, specific nucleic acid probes, and rolling-circle amplification (RCA) technology, developed by Söderberg *et al.* (2006), for detecting PPIs and usually uses two primary antibodies derived from different species (Miki* et al.* 2023*a*,*b*). Secondary antibodies labeled with oligonucleotides (PLA probes), such as anti-rabbit and anti-mouse antibodies, bind to the primary antibodies of different animal species. The connector oligo hybridizes only when the two PLA probes are close to each other (less than 40 nm), and the PLA probe acts as a primer for DNA polymerase to amplify DNA using RCA. Finally, a fluorescent-labeled oligonucleotide probe hybridizes to the amplified complementary sequence and is visualized as a fluorescent spot (PLA signal). In the aforementioned *GR* in the nuclei of MDA-MB-231 cells, red dots indicating dimers were detected by PLA, and it was confirmed that GR dimers were formed (Fig. 6).

**Figure 7:**
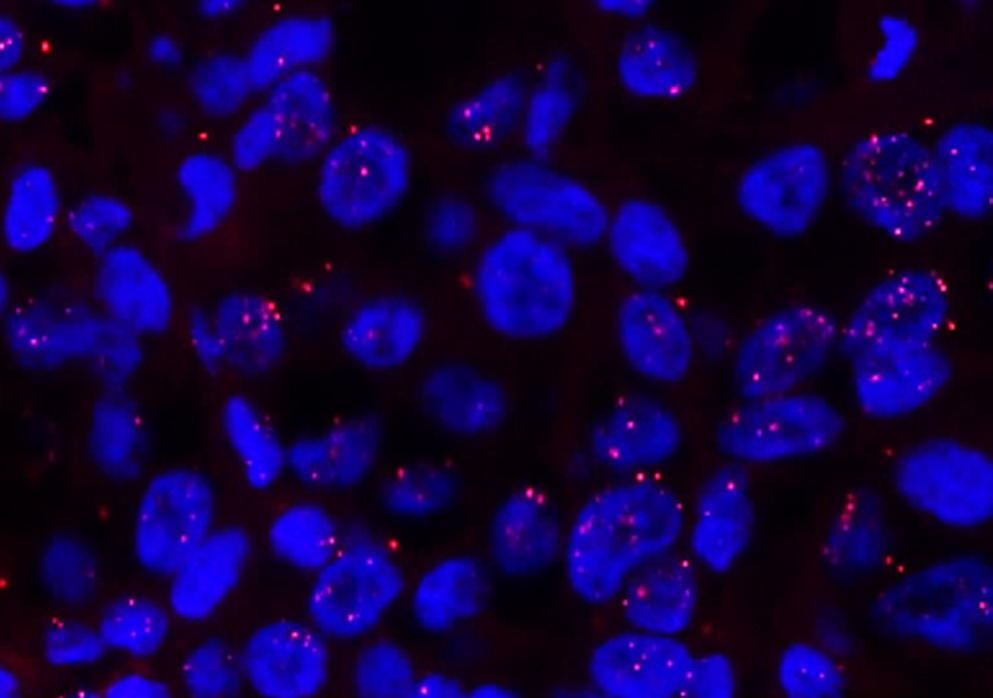
*In situ* proximity ligation assay of ERα in breast cancer tissue. *In situ* proximity ligation assay (PLA) of ERα in invasive ductal carcinoma is demonstrated. The mouse monoclonal 6F11 antibody and rabbit monoclonal antibody SP1 were used for PLA.

We previously detected ERα homodimers on breast cancer FFPE tissue by PLA using antibodies against ERα, rabbit monoclonal SP1, and mouse monoclonal 6F11 (Iwabuchi *et al.* 2017) (Fig. 7). In PLA using FFPE tissue in 25 breast cancer cases, the ERα PLA score was positively correlated with the ERα or PgR immunohistochemistry score. Furthermore, using the ERα antibody SP1 and the mouse monoclonal ERβ antibody 14C3, we revealed that ERα/ERβ heterodimers and ERα homodimers coexist in the same breast cancer case (Iwabuchi *et al.* 2017). Snell *et al.* (2018) investigated the significance of the interaction between ER (rabbit monoclonal SP1) and PgR isoform B (PRB) (mouse monoclonal 3E11) using PLA in 229 breast cancer cases. Although the ER-PRB status independently predicted recurrence, the PgR status assessed solely by immunohistochemistry was not. Furthermore, a low ER-PRB status was predictive of relapse with adjuvant aromatase inhibitor therapy. Using bimolecular fluorescence complementation analysis *in vitro*, we previously reported that PgR and Grb2 interact in breast cancer cells through the Grb2-SH3 domain (Wittayavimol *et al.* 2024). Furthermore, we performed PLA analysis using a mouse monoclonal PgR antibody and rabbit monoclonal Grb2 antibody (Y237) and Grb2 immunohistochemical analysis in 43 breast cancer cases (Wittayavimol *et al.* 2024). Immunohistochemistry revealed a negative relationship between Grb2 status and lymph node metastasis, whereas in PLA, a negative relationship between PgR-Grb2 status and lymphatic invasion and stage was also observed. These findings suggest that the PLA can provide additional information on hormonal signals in pathological tissue specimens. In steroid hormone signaling research using PLA, *in vitro* analysis using cultured cells is still mainstream (Iwabuchi *et al.* 2022), but clinical significance can be determined by visualizing these signals in pathological FFPE tissues.

## Visualization of hormone receptor signals: detection of receptor-DNA binding

Southwestern histochemistry, established by Koji *et al.* (1994), visualizes the localization of DNA-binding proteins in tissue specimens. In Southwestern histochemistry, an oligonucleotide probe complementary to the DNA-binding site of the target protein, labeled with a thymine-thymine (T-T) dimer or digoxigenin (DIG), is first reacted with the tissue. The probe that reacts with the tissue is detected immunohistochemically using anti-T-T dimer or anti-DIG antibodies. In the binding analysis of ER to response elements, the palindromic estrogen response element (ERE) (5’-GATCCAGGTCACAGTGACCTGGATC-3’) of the chicken vitellogenin gene is used as a probe (Koji *et al.* 1994). Dimeric ER binds to the ERE present in the promoter region of estrogen-responsive genes, indicating ER activation. In the PLA described in the previous section, receptor dimers are directly detected using antibodies, whereas southwestern histochemistry uses DNA probes to detect ERE-binding proteins, which are ER dimers. In PLA, the homodimers and heterodimers of hormone receptors can be distinguished using specific antibodies against these hormone receptors. In contrast, southwestern histochemistry does not require the preparation of hormone receptor-specific antibodies but cannot distinguish between hormone receptor dimer patterns.

**Figure 8:**
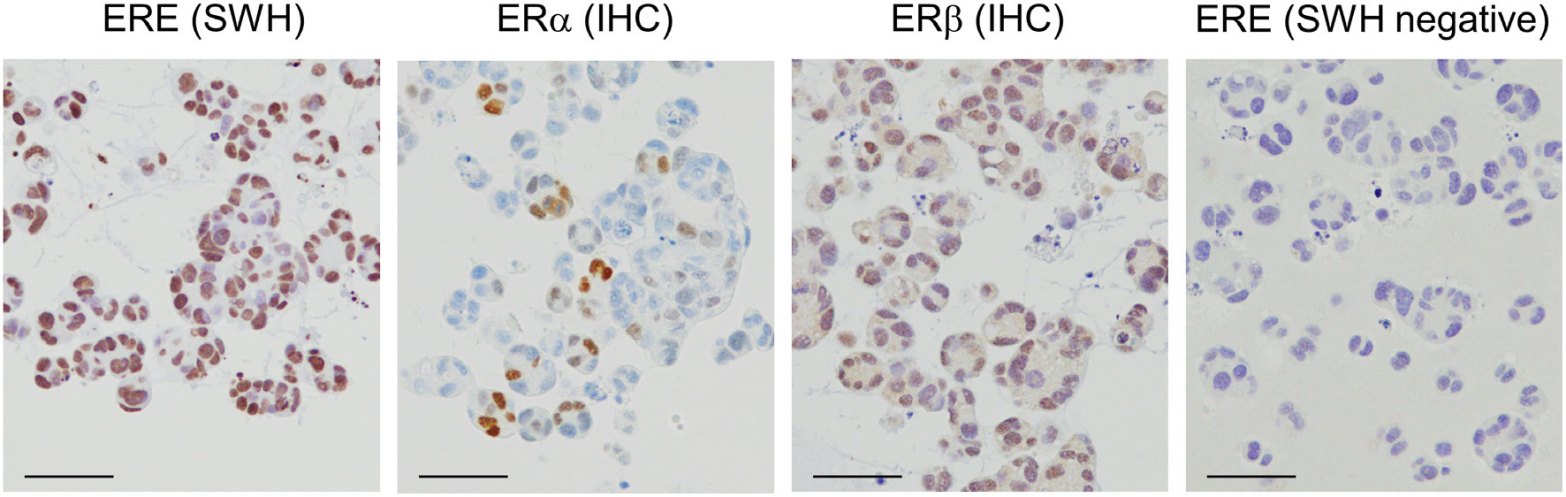
Southwestern histochemistry of estrogen response element binding protein in endometrial cancer cell lines. Southwestern histochemistry (SWH) of estrogen response element (ERE) binding protein and immunocytochemistry (ICC) of estrogen receptor (ER) α and ERβ in Ishikawa (three-dimensional culture) are shown. The SWH mutant probe was used as a negative control (SWH-negative).

Figure 8 shows the southwestern histochemistry of the ERE using the endometrial cancer cell line Ishikawa. The specimens used were Ishikawa three-dimensionally cultured FFPE cells. The ERE probe described above was DIG tail-labeled, and an anti-DIG antibody was used for immunohistochemical detection. In the Ishikawa cells, signals indicating ERE binding were observed in the nuclei of many cells. Immunohistochemistry showed that many cells in Ishikawa were negative for ERα but positive for ERβ. Therefore, the southwestern histochemistry results may indicate an ERβ reaction. Simultaneously, in southwestern histochemistry, using a mutant ERE probe to confirm that no reaction occurred is necessary. Southwestern histochemistry using FFPE tissue has been reported for ER signaling in mouse ovaries (Hishikawa *et al.* 2003, Shin *et al.* 2002). In these reports, the localization pattern of ERE detected by southwestern histochemistry was consistent with that of ERα and ERβ detected by immunohistochemistry. Analyses of FFPE tissues of the testes and ovaries from HMGB2 knockout mice were reported to reveal a decrease in estrogen response element-binding sites using southwestern histochemistry (Sugita *et al.* 2021, Yamaguma *et al.* 2022). Because southwestern histochemistry cannot determine the pattern of protein complexes that bind to DNA, evaluation in conjunction with expression/localization analysis using immunohistochemistry will lead to a more detailed elucidation of hormone signals.

## Future perspectives

Mass spectrometry imaging (MSI) using matrix-assisted laser desorption/ionization (MALDI) mass spectrometry displays the intensity distribution of arbitrary peaks from mass spectra obtained by performing mass spectrometry directly on the surface of frozen tissue sections. Steroid hormones, which are small molecules, have low ionization efficiency and are easily affected by high-abundance contaminants; therefore, derivatization reagents are sprayed over the entire section as a sample pretreatment to improve sensitivity (on-tissue derivatization) (Shariatgorji *et al.* 2015, Li *et al.* 2020). Immunohistochemistry using a specific antibody for *CYP11B2*, an aldosterone synthase, revealed that *CYP11B2*-positive cells exist in clusters rather than diffusely in normal human adrenal glands and the concept of an aldosterone-producing cell cluster (APCC) (Nishimoto *et al.* 2010). The MSI of cortisol and aldosterone in normal adrenal glands showed a diffuse distribution of cortisol, whereas aldosterone was concentrated in the APCC, which was positive for *CYP11B2* immunoreactivity (Takeo *et al.* 2019). MSI allows the spatial visualization of the distribution of unlabeled steroid hormones in tissue sections. Visualization of steroid hormones using MSI requires improvements in quantitative performance, sensitivity, and resolution; however, MSI has also been used to elucidate the physiological functions and pathology of hormone-related tissues and their disorders (Sugiura *et al.* 2018, Li *et al.* 2020).

Laser capture microdissection (LCM) is a method of obtaining tissue in a region of interest by physically cutting it out using a laser and then using RNA extracted from that region for downstream analyses, such as quantitative PCR and RNA sequence analysis (Miki *et al.* 2007, Miki *et al.* 2010, Nichterwitz *et al.* 2016). Because the laser diameter in LCM analysis is limited, the resolution is limited to separating the cancer from the stroma or cutting out a single slice of the glandular cavity. In single-cell gene expression analyses, such as conventional single-cell RNA sequencing, tissue location information is lost. In recent years, single-cell spatial transcriptome analysis has been performed at the single-cell level while preserving spatial information on tissue sections. Spatial and single-cell transcriptome analyses of estrogen responsiveness have been performed in a breast cancer patient-derived xenograft (PDX) model (Yoshitake *et al.* 2024). Frozen tissues from an established ER-positive PDX model were used in this study. Tissue mapping of gene transcription revealed four spatially distinct populations with unique genetic signatures that contributed to cancer progression. These four populations exhibit estrogen-responsive, proliferative, hypoxia-induced, and inflammation-related characteristics. This gene transcriptional distribution finding suggests that a ‘proliferative’ rather than an ‘estrogen-responsive’ population is important for estrogen-dependent tumor growth. By mapping gene transcription profiles in pathological tissues, it is possible to comprehensively explore differences in the expression patterns of hormone receptor-responsive genes among components of the tumor tissue, such as normal epithelial cells, cancer cells, and fibroblasts (Leach *et al.* 2022). The use of frozen tissue for sequencing is considered the gold standard because formalin causes strand breaks in the RNA and impairs its interactions with other molecules.

## Conclusion

Histological analyses of each process in the steroid hormone signaling pathway are summarized in Fig. 1. Classical immunohistochemistry has demonstrated many processes involved in steroid hormone signaling in FFPE tissues. Furthermore, PLA, which combines antibody technology and RCA, enables the detection of PPIs that cannot be detected using immunohistochemistry. If suitable antibodies for immunohistochemistry are not available, *in situ* hybridization, which is not discussed in this review, may be an effective tool. The binding of DNA to receptor dimers, which is difficult to detect using antibody techniques such as immunohistochemistry, was revealed by southwestern histochemistry using response element-specific probes in FFPE tissues. Although limited to frozen tissues, MSI has also been developed to map hormones onto tissues, which is expected to advance our understanding of steroid synthesis.

Since pathological tissue specimens cannot show dynamic changes, they are undeniably ‘snapshots’ rather than ‘movies.’ However, by comparing the preoperative specimen (i.e., biopsy sample) with the surgical specimen from the same case, the effect of neoadjuvant therapy can be evaluated. Furthermore, retrospective studies using past specimens can clarify factors associated with prognosis and recurrence. Innovations in molecular biology have enabled the overlay of gene expression profiles onto the spatial information of FFPE tissues. By utilizing the characteristics of FFPE in pathological tissues, histochemical analysis can be a useful tool for both steroid hormone signaling and translational medicine.

## Declaration of interest

The authors declare that there is no conflict of interest that could be perceived as prejudicing the impartiality of the research reported.

## Funding

This work did not receive any specific grant from any funding agency in the public, commercial, or not-for-profit sector.
